# Digital postpartum hemorrhage management device (DPHMD)

**DOI:** 10.1186/s12884-019-2601-3

**Published:** 2019-11-26

**Authors:** Derartu D. Tekela, Abeba G. Asmare, Birhan M. Gebremariam, Christian A. Assegahegn, Kidist D. Wami, Hundessa D. Nemomssa, Gizeaddis L. Simegn

**Affiliations:** School of Biomedical Engineering, Jimma Institute of Technology, Jimma University, Jimma, Ethiopia

**Keywords:** Postpartum hemorrhage, Blood loss, Blood pressure, Diagnosis, Fluid delivery, Maternal mortality

## Abstract

**Background:**

Primary postpartum hemorrhage (PPH) is an obstetric emergency caused by excessive blood loss that occurs most commonly after the placenta is delivered. PPH can lead to volume depletion, hypovolemic shock, anemia, and it is the leading cause of maternal mortality worldwide. With 470 deaths per 100,000 live births, the maternal mortality ratio in Ethiopia is one of the highest in the world. It is estimated that 94% of births occur at home in Ethiopia and that 10% of maternal deaths are attributed to PPH. Currently, physicians use visual estimation to calculate blood loss and provide fluid during delivery. This traditional method is subjective and generally inaccurate.

**Method:**

In this project, after delivery blood loss measurement system integrated with fluid delivery and vital sign monitoring method is proposed. The collection and measurement system collects blood loss after delivery and measures the amount of blood loss. The management system continuously monitors the mother’s heart rate and blood pressure. These vital sign values are integrated with the measured blood loss to estimate the amount of IV fluid required to be delivered for the mother. The rate of IV fluid delivery is regulated by a flow rate sensor and solenoid valve.

**Results:**

The prototype was built and undergone through different tests and iterations. The proposed device was tested for accuracy, cost effectiveness and ease to use. 91.28% accuracy has been achieved and the prototype was built with less than 210 USD.

**Conclusion:**

The proposed design allows physicians, especially those in low resource setting, to estimate blood loss and deliver fluid accurately. This helps to reduce maternal mortality rate that may occur due to postpartum hemorrhage.

## Background

Primary postpartum hemorrhage (PPH) is defined as blood loss of 500 ml after vaginal delivery and above or 1000 ml of blood loss after caesarean section within the first 24 h [1, 2]. It is the most common cause of premature mortality of women worldwide. PPH is dangerous and life-threatening and can also lead to long-lasting health effects, including severe anemia [3]. According to the 2013 World Health Statistics, the maternal mortality rate in low income countries were 410/100,000 live births [4]. The majority of maternal deaths occurred mainly in Asian and African countries [5]. Major causes of maternal deaths are similar across low income countries, often obstetric in origin including hemorrhage, hypertensive diseases and maternal infections [4, 6–12]. 94% of births in Ethiopia are estimated to occur at home and 10% of maternal deaths are attributed to PPH [13].

Uterine atony, or lack of effective contraction of the uterus, is the most common cause of PPH [3] followed by infection, subinvolution of the placental site, and inherited coagulation deficits [14–17]. The majority of these fatal obstetric complications occur during labor and immediately after birth. In the low income countries, more than three-quarters of maternal deaths due to the direct obstetric causes occur during and after birth [4, 18, 19]. Organized diagnosis and management of PPH, including administration of uterotonic agents [20], controlled cord traction, and uterine massage after delivery of the placenta, are required to avoid maternal death.

The high frequency of PPH in the developing world is due to the lack of diagnosis and management methods as well as medications used in the active management of the third stage. Lack of experienced caregivers who can manage PPH and lack of blood transfusion services, anesthetic services, and operating capabilities also play a role.

A well-defined stepwise approach is recommended for treatment of uterine atony, including drugs and mechanical interventions, followed by surgery as a last intervention [3, 21, 22]. The first diagnosis of PPH is performed by observing the amount of blood loss and the patient’s clinical status. The amount of blood loss, the patient’s level of consciousness and vital signs are continually assessed. Photospectometry is the gold standard blood loss measurement technique due to its accuracy. However, this technique is complicated, costly and impractical. It cannot be applied at all levels of healthcare and is more suitable for clinical research [23–25]. Weighed soaked swabs or drapes after delivery are also used for early detection of PPH [26]. However, this method substantially increases the workload of physicians and may not be suitable in a busy hospital setting. Bakri balloon [27], arterial embolization [28] and absorbable sutures [29] are other methods used to manage and reduce PPH. However, most of the techniques are either expensive and complex to apply in low resources settings or are associated with complications.

Currently, in low resource settings blood loss during delivery is estimated manually through visual inspection. Visual estimation of blood loss is subjective and generally inaccurate. Studies have shown that, irrespective of physicians’ experience or skill level, visual estimation of PPH could result 25–89% measurement error [24].

In this project digitalized postpartum hemorrhage management device (DPHMD) is proposed to collect and measure blood loss, monitor vital signs and estimate the amount of IV fluid required to manage PPH at early stage. The proposed method can be used as a decision support system for physicians especially in low resource settings where both the expertise and medical devices are in scarce.

## Methods

### Proposed design

**Fig. 1:**
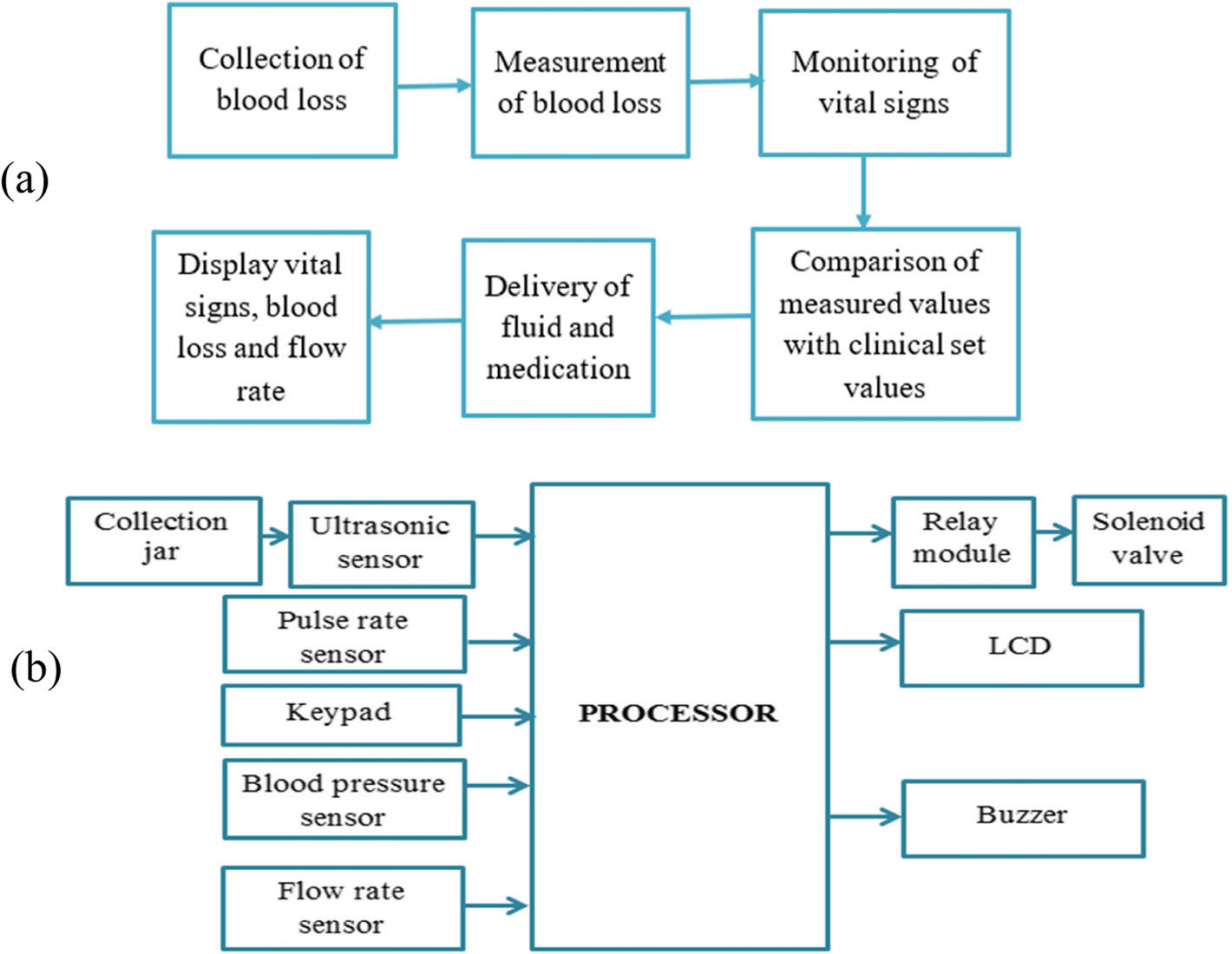
Functional and general block diagram of DPHMD

**Fig. 2:**
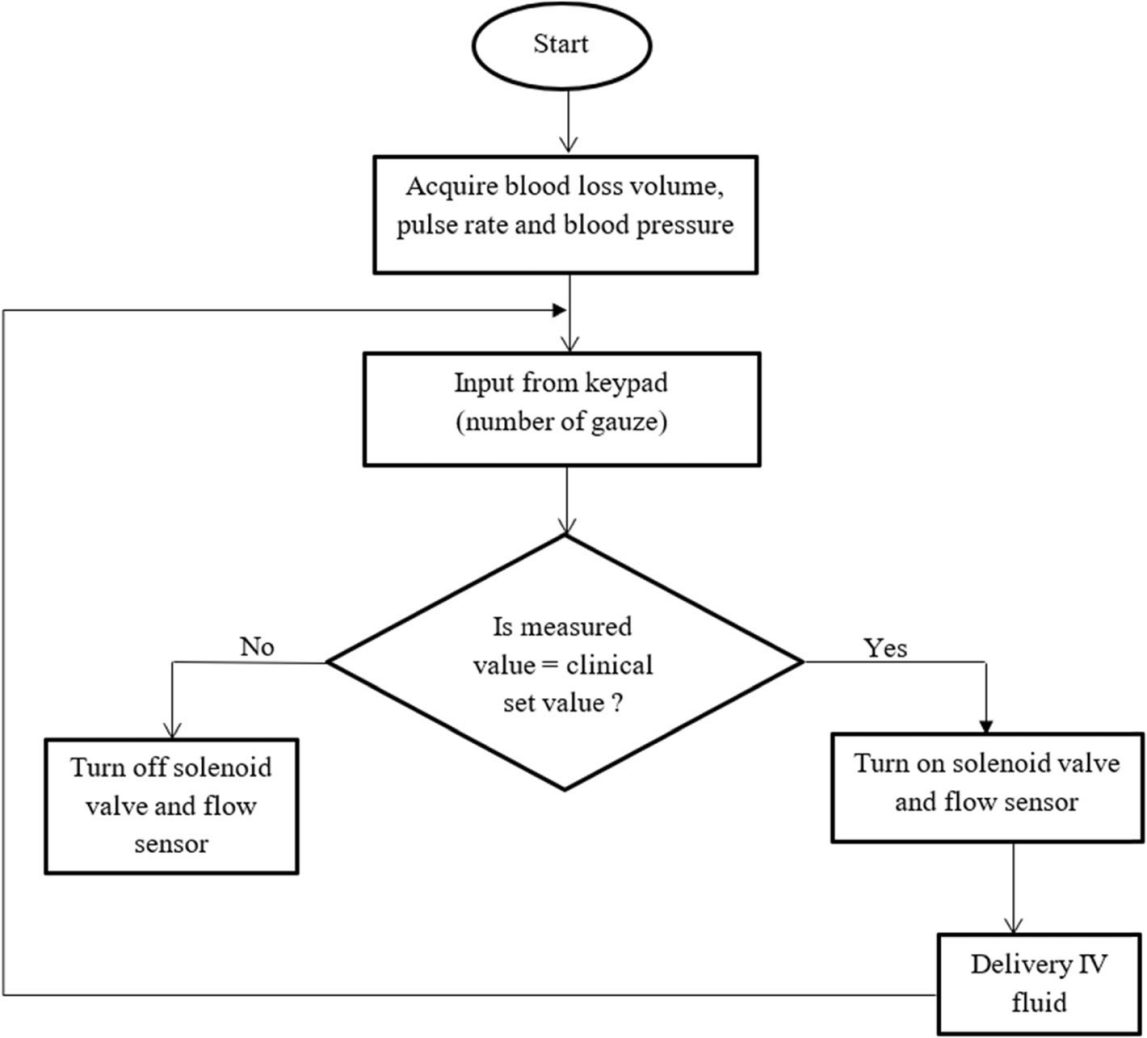
Flow chart of IV fluid delivery controller

The Proposed solution includes blood loss collection and measurement system, vital sign monitor (pulse rate and blood pressure), processor unit (Arduino microcontroller), low rate monitor and regulator, display and alarm system. Inputs from the blood loss measurement system, vial sign monitor as well as the number of gauze used from the key-pad are used to estimate the recommended IV fluid to be delivered. The alarm is used to notify the physicians in case of severe conditions. Under-buttock drape, which allows the blood loss to enter to the collection jar without loss, was constructed from locally available material. Ultrasonic sensor is used to measure the volume of blood collected in the jar. Figure 1 show the functional block diagram and general block diagram of our proposed design. The solenoid valve controls the amount of fluid to be delivered to the patient. Solenoid valve and flow sensor will stay on until enough fluid is delivered. The flow rate will be used to calculate the amount of fluid delivered. If the measured value is larger than the clinical set value the solenoid value will be turned off automatically to prevent excess medication. Figure 2 show the slow chart of fluid medication controller.

## Results

### Final design

**Fig. 3:**
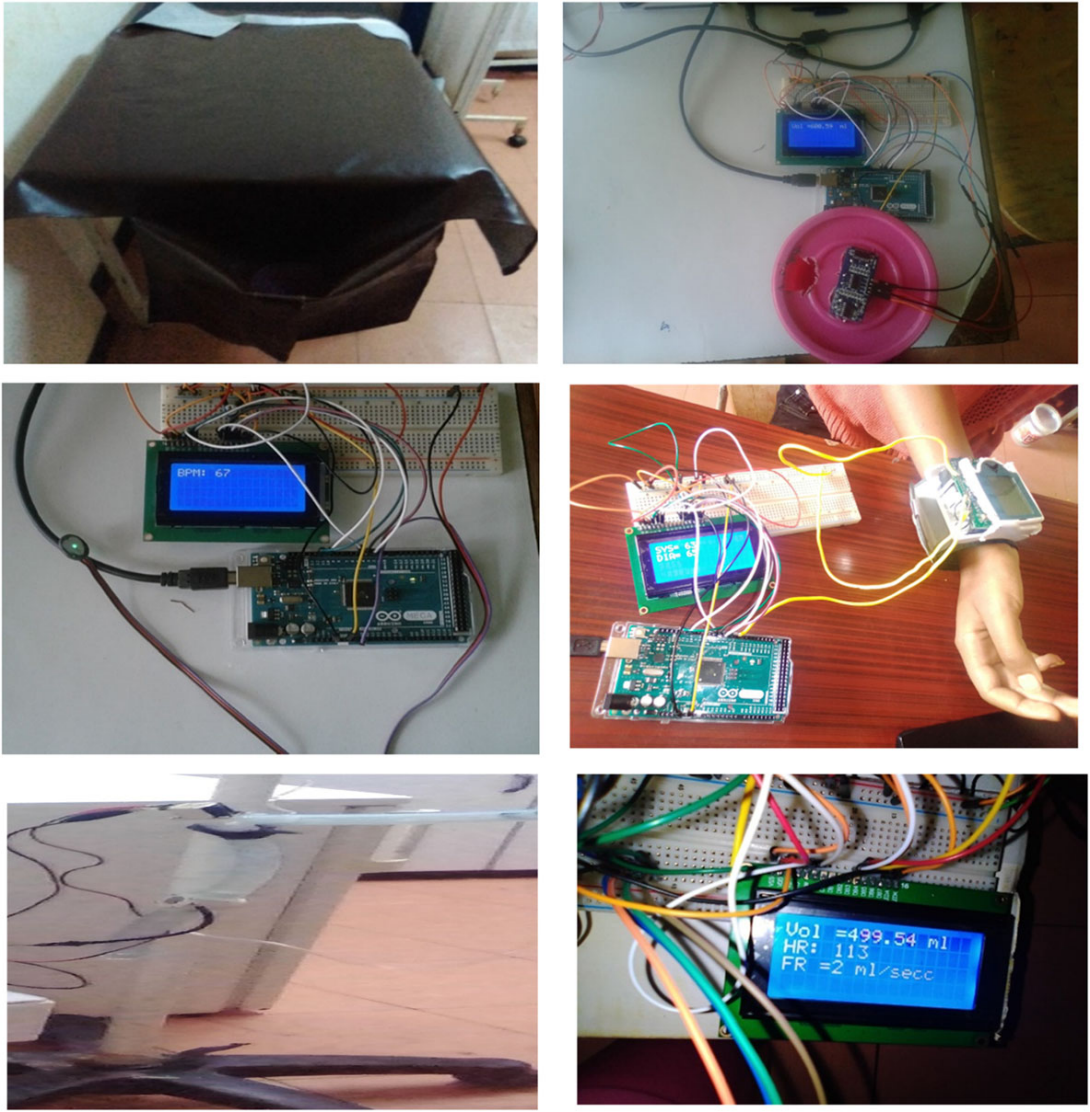
Components of DPHMD design

Different prototype iterations has been conducted to modify our design. Figure 3 shows parts of the Final design: (Left to Right: Top to Bottom) under-buttock drape for smooth flow of blood to the jar, collection jar and an ultrasonic sensor to collect and measure blood loss, heart rate and pressure sensor to measure the two parameters the two vital signs, a flow sensor and a solenoid valve to indicate flow rate and allow one directional flow of IV fluid, respectively and a display system.

**Fig. 4:**
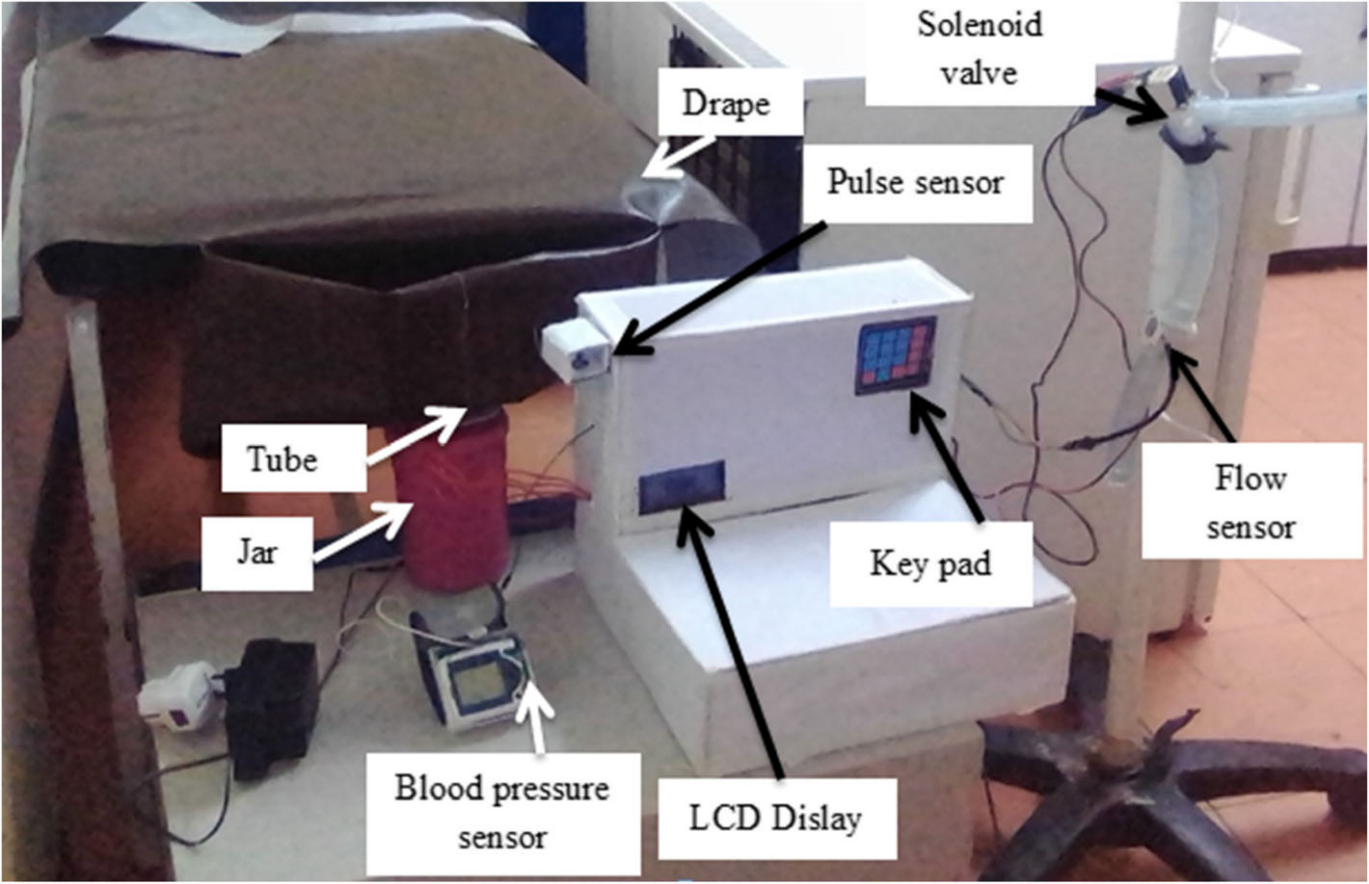
The final design of DPHMD and its parts

The following components has been used in the final design: HC-SR04 Ultrasonic Sensor, Arduino Uno, Flow Sensor YF-S201, Liquid crystal display (LCD), Buzzer, 4 × 4 Keypad, Plastic solenoid valve, Pulse sensor, Blood pressure sensor, Plastic jar, Drape, Plastic tube, Resistors, Potentiometer, Jumper wires, and USB cable. Figure 4 shows the final design of DPHMD.

Several tests and iteration were used in order to verify whether the design criteria and specification were fulfilled. Accuracy, cost effectiveness and easy to use are the parameters tested. Table 1 shows the test results.

**Table 1:** Test methods and results

Criteria	Input	Method	Iterations	Result
Accuracy	Blood volume	By using known amount of water	Five times	91.28%
	Heart rate	By using manual counting method	Five times	
	Blood pressure	By using existing method	Five times	
Cost effectiveness	Market analysis	Total components cost	_	210 USD
Easy to use	Operating procedure	30 min training for Physicians	–	Simple

## Discussion

The prevalence of PPH is disproportionately higher in low resource settings where there is limited access to skilled medical care and safe blood supplies. Despite the fact that it is largely preventable, by improving the quality of care, postpartum hemorrhage is the most common and most deadly form of obstetric bleeding [9]. Initial treatment of PPH includes uterotonic medications such as oxytocin and misoprostol plus bimanual massage. However, proper collection and estimation of blood loss is required to manage PPH. This study presented a method for diagnosis and management of PPH digitally.

Visual estimation of blood loss including weighing of soaked pad, which is the current method for estimating amount of blood loss in low resource settings, is generally inaccurate and may result misdiagnosis. The *calibrated* blood collection *drape* was also proposed to assist in estimating postpartum blood loss in low-resource settings [30, 31]. However, estimating blood loss alone may not give enough information about the status of the patient. Blood pressure and heart rate monitoring is key to hemodynamic assessment, with thresholds for systolic blood pressure (SBP) and pulse used in clinical trigger or early warning systems to prompt intervention [23, 32]. Shock index, which is the ratio of heart rate and systolic pressure, are also used to predict blood loss in patients with PPH [33, 34]. However, using vital signs in isolation may lead to inaccurate decision since vital sign change due to PPH can be masked by the hemodynamic changes of pregnancy [35]. Our method provides both measurement of blood loss as well as vital signs monitoring to detect and manage PPH.

Every design is preferable to be easy to use, accurate and low cost. Our design is simple and user friendly. The traditional manual PPH managing method is digitalized by incorporating vital signs monitoring and blood loss measurement in one system. This helps physicians to easily adapt the digitalized system with a minimum training. The prototype costs only 210 USD making it affordable for low resource settings. The accuracy of the designed system is inspected by performing different tests with the assistance of obstetric physicians. The blood loss collection and measurement system, vital signs measurement and flow rate sensor were tested. A total of 91.28% accuracy has been achieved with five iterations on different subjects. The blood loss estimation was 98% accurate which is much better than the accuracy of visual estimation, that was found to be 25–89% accurate as reported in many studies [24–26, 36, 37]. The proposed design provides high level of safety. It is free from electrical shock, contamination or infections and any type of hazardous radiation exposure.

## Conclusion

In order to prevent complications, effective management of postpartum hemorrhage plays a huge role in treating and saving mothers suffering from PPH. Our Digitalized postpartum management device can be used as a decision support system for physicians by determining the amount of blood loss and the patient’s level of consciousness through vital signs continuous monitoring. The prototype was built and undergo through different tests and iterations and it is 91.28% accurate. The proposed method will have a great impact in low resource settings where both the expertise and means is in scarce.

## Data Availability

Not applicable.

## Abbreviations

DPHMD: Digital Postpartum Hemorrhage Management Device
LCD: Liquid crystal display
PPH: Postpartum Hemorrhage
SBP: Systolic Blood Pressure

## References

[1] Edhi MM, Aslam HM, Naqvi Z, Hashmi H (2013). Post partum hemorrhage: causes and management. BMC Res Notes.

[2] Weeks A (2015). The prevention and treatment of postpartum haemorrhage: what do we know, and where do we go to next?. BJOG Int J Obstet Gynaecol.

[3] Herrick T, Mvundura M, Burke TF, Abu-Haydar E (2017). A low-cost uterine balloon tamponade for management of postpartum hemorrhage: modeling the potential impact on maternal mortality and morbidity in sub-Saharan Africa. BMC Pregnancy Childbirth.

[4] Berhan Y, Berhan A (2014). Review of maternal mortality in Ethiopia: a story of the past 30 years. Ethiop J Health Sci.

[5] Hogan MC, Foreman KJ, Naghavi M, Ahn SY, Wang M, Makela SM (2010). Maternal mortality for 181 countries, 1980–2008: a systematic analysis of progress towards Millennium Development Goal 5. Lancet.

[6] Alvarez JL, Gil R, Hernandez V, Gil A (2009). Factors associated with maternal mortality in sub-Saharan Africa: an ecological study. BMC Public Health.

[7] Goldenberg RL, McClure EM, Saleem S (2018). Improving pregnancy outcomes in low- and middle-income countries. Reprod Health.

[8] Khan KS, Wojdyla D, Say L, Gulmezoglu AM, Van Look PF (2006). WHO analysis of causes of maternal death: a systematic review. Lancet.

[9] Say L, Chou D, Gemmill A, Tuncalp O, Moller AB, Daniels J (2014). Global causes of maternal death: a WHO systematic analysis. Lancet Glob Health.

[10] Rahman MM, Abe SK, Rahman MS, Kanda M, Narita S, Bilano V (2016). Maternal anemia and risk of adverse birth and health outcomes in low- and middle-income countries: systematic review and meta-analysis. Am J Clin Nutr.

[11] Bauserman M, Lokangaka A, Thorsten V, Tshefu A, Goudar SS, Esamai F (2015). Risk factors for maternal death and trends in maternal mortality in low- and middle-income countries: a prospective longitudinal cohort analysis. Reprod Health.

[12] McClure EM, Garces A, Saleem S, Moore JL, Bose CL, Esamai F (2018). Global network for Women's and Children's Health Research: probable causes of stillbirth in low- and middle-income countries using a prospectively defined classification system. BJOG.

[13] Hddis M, Woyessa A (2012). Prevention of Postpartum Hemorrhage in Rural Ethiopia (SURE policy brief).

[14] Lin L, Chen YH, Sun W, Gong JJ, Li P, Chen JJ (2019). Risk factors of obstetric admissions to the intensive care unit: an 8-year retrospective study. Medicine..

[15] Changede P, Chavan N, Raj N, Gupta P (2019). An observational study to evaluate the maternal and Foetal outcomes in pregnancies complicated with jaundice. J Obstetr Gynaecol India.

[16] Joseph CM, Bhatia G, Abraham V, Dhar T (2018). Obstetric admissions to tertiary level intensive care unit - prevalence, clinical characteristics and outcomes. Indian J Anaesthesia.

[17] Gillissen A, van den Akker T, Caram-Deelder C, Henriquez D, Bloemenkamp KWM, de Maat MPM (2018). Coagulation parameters during the course of severe postpartum hemorrhage: a nationwide retrospective cohort study. Blood Adv.

[18] Li XF, Fortney JA, Kotelchuck M, Glover LH (1996). The postpartum period: the key to maternal mortality. Int J Gynaecol Obstetr.

[19] Organization WH (1994). Mother baby package, Implementing Safe Motherhood in Countries. Maternal and Safe Motherhood Programme.

[20] Stanton CK, Newton S, Mullany LC, Cofie P, Agyemang CT, Adiibokah E (2012). Impact on postpartum hemorrhage of prophylactic administration of oxytocin 10 IU via UnijectTM by peripheral health care providers at home births: design of a community-based cluster-randomized trial. BMC Pregnancy Childbirth..

[21] Lalonde A (2012). Prevention and treatment of postpartum hemorrhage in low-resource settings. Int J Gynaecol Obstetr.

[22] Organization WH. WHO recommendations for the prevention and treatment of postpartum haemorrhage. Geneva: World Health Organization; 2012. https://www.ncbi.nlm.nih.gov/pubmed/23586122. 23586122

[23] Schorn MN (2010). Measurement of blood loss: review of the literature. J Midwif Womens Health.

[24] Lertbunnaphong T, Lapthanapat N, Leetheeragul J, Hakularb P, Ownon A (2016). Postpartum blood loss: visual estimation versus objective quantification with a novel birthing drape. Singap Med J.

[25] Patel A, Goudar SS, Geller SE, Kodkany BS, Edlavitch SA, Wagh K (2006). Drape estimation vs. visual assessment for estimating postpartum hemorrhage. Int J Gynaecol Obstetr.

[26] Al Kadri HM, Al Anazi BK, Tamim HM (2011). Visual estimation versus gravimetric measurement of postpartum blood loss: a prospective cohort study. Arch Gynecol Obstet.

[27] Aibar L, Aguilar MT, Puertas A, Valverde M (2013). Bakri balloon for the management of postpartum hemorrhage. Acta Obstet Gynecol Scand.

[28] Kim T-H, Lee H-H, Kim J-M, Ryu A-L, Chung S-H, Seok LW (2013). Uterine artery embolization for primary postpartum hemorrhage. Iran J Reprod Med.

[29] Al Riyami N, Hui D, Herer E, Nevo O (2011). Uterine compression sutures as an effective treatment for postpartum hemorrhage: case series. AJP Rep.

[30] Tixier H, Boucard C, Ferdynus C, Douvier S, Sagot P (2011). Interest of using an underbuttocks drape with collection pouch for early diagnosis of postpartum hemorrhage. Arch Gynecol Obstet.

[31] Toledo P, McCarthy RJ, Hewlett BJ, Fitzgerald PC, Wong CA (2007). The accuracy of blood loss estimation after simulated vaginal delivery. Anesthesia and analgesia.

[32] El Ayadi AM, Nathan HL, Seed PT, Butrick EA, Hezelgrave NL, Shennan AH (2016). Vital Sign Prediction of Adverse Maternal Outcomes in Women with Hypovolemic Shock: The Role of Shock Index. PLoS One.

[33] Le Bas A, Chandraharan E, Addei A, Arulkumaran S (2014). Use of the “obstetric shock index” as an adjunct in identifying significant blood loss in patients with massive postpartum hemorrhage. Int J Gynecol Obstet.

[34] Nathan HL, El Ayadi A, Hezelgrave NL, Seed P, Butrick E, Miller S (2015). Shock index: an effective predictor of outcome in postpartum haemorrhage?. BJOG.

[35] Bonanno FG (2012). Hemorrhagic shock: the “physiology approach”. J Emergencies Trauma Shock.

[36] Prasertcharoensuk W, Swadpanich U, Lumbiganon P (2000). Accuracy of the blood loss estimation in the third stage of labor. Int J Gynaecol Obstetr.

[37] Yoong W, Karavolos S, Damodaram M, Madgwick K, Milestone N, Al-Habib A (2010). Observer accuracy and reproducibility of visual estimation of blood loss in obstetrics: how accurate and consistent are health-care professionals?. Arch Gynecol Obstet.

